# Identification of SYS1 as a Host Factor Required for Shiga Toxin-Mediated Cytotoxicity in Vero Cells

**DOI:** 10.3390/ijms22094936

**Published:** 2021-05-06

**Authors:** Chisato Sakuma, Tsuyoshi Sekizuka, Makoto Kuroda, Kentaro Hanada, Toshiyuki Yamaji

**Affiliations:** 1 Department of Biochemistry and Cell Biology, National Institute of Infectious Diseases, 1-23-1 Toyama, Shinjuku-ku, Tokyo 162-8640, Japan; sakumac@nih.go.jp (C.S.); hanak@nih.go.jp (K.H.); 2 Pathogen Genomics Center, National Institute of Infectious Diseases, 1-23-1 Toyama, Shinjuku-ku, Tokyo 162-8640, Japan; sekizuka@nih.go.jp (T.S.); makokuro@nih.go.jp (M.K.)

**Keywords:** glycosphingolipid, Vero, CRISPR/CAS9, genome-wide screening, Shiga toxin, SYS1

## Abstract

Shiga toxin (STx) or Vero toxin is a virulence factor produced by enterohemorrhagic *Escherichia coli*. The toxin binds to the glycosphingolipid globotriaosylceramide (Gb3) for its entry, and causes cell death by inhibiting ribosome function. Previously, we performed a loss-of-function screen in HeLa cells using a human CRISPR knockout (KO) library and identified various host genes required for STx-induced cell death. To determine whether this library targeted to the human genome is applicable to non-human primate cells and to identify previously unrecognized factors crucial for STx-induced cell death, we herein performed a similar screen in the African green monkey kidney-derived Vero C1008 subline. Many genes relevant to metabolic enzymes and membrane trafficking were enriched, although the number of enriched genes was less than that obtained in the screening for HeLa cells. Of note, several genes that had not been enriched in the previous screening were enriched: one of these genes was *SYS1*, which encodes a multi-spanning membrane protein in the Golgi apparatus. In *SYS1* KO Vero cells, expression of Gb3 and sphingomyelin was decreased, while that of glucosylceramide and lactosylceramide was increased. In addition, loss of *SYS1* inhibited the biosynthesis of protein glycans, deformed the Golgi apparatus, and perturbed the localization of *trans*-Golgi network protein (TGN) 46. These results indicate that the human CRISPR KO library is applicable to Vero cell lines, and SYS1 has a widespread effect on glycan biosynthesis via regulation of intra-Golgi and endosome–TGN retrograde transports.

## 1. Introduction

Shiga toxin (STx) is a key virulence factor produced by enterohemorrhagic *Escherichia coli* (EHEC) as well as *Shigella dysenteriae* serotype 1 [1,2]. STx causes hemorrhagic colitis with potentially lethal complications of hemolytic uremic syndrome and encephalopathy [3,4]. STx is composed of a catalytic A-subunit that inactivates ribosomes to inhibit protein synthesis and a B-subunit homo-pentamer that binds to the target cells [5,6]. The receptor for STx is globotriaosylceramide (Gb3), a type of glycosphingolipid (GSL) [7,8]. After binding to Gb3 on the cell surface, STx is internalized and transported from endosomes to the endoplasmic reticulum via the *trans*-Golgi network (TGN)/the Golgi apparatus [9]. Then, the A-subunit is translocated to the cytosol, where it inhibits ribosomes and causes cell death [10,11].

Gb3 is synthesized by continuous glycosylations of ceramide. Ceramide is converted to glucosylceramide (GlcCer) by UDP-glucose ceramide glucosyltransferase (UGCG) on the cytosolic face of the *cis* Golgi [12,13]. After traversing the Golgi membrane, GlcCer is converted to lactosylceramide (LacCer) mainly by β1,4 galactosyltransferase 5 (B4GalT5) in the luminal side of the Golgi [14,15]. LacCer is converted to several GSLs, one of which is Gb3 synthesized by α1,4 galactosyltransferase (A4GalT) at the TGN [16,17,18]. Ceramide is also converted to sphingomyelin in the *trans*-Golgi regions after transportation from the endoplasmic reticulum to the Golgi apparatus by the ceramide transport protein CERT in a non-vesicular manner [19,20].

Proper glycosylation of GSLs requires post-translational regulation of GSL enzymes as well as GSL transport. For example, the conserved oligomeric Golgi (COG) complex, a multisubunit tethering complex found in the Golgi apparatus, maintains the localization of Golgi-resident glycan enzymes by retrograde trafficking [21]. Defects in COG subunits lead to congenital disorders of glycosylation-type II, a group of inherited metabolic disorders defective in protein glycosylation [22,23].

We and other groups have employed CRISPR genome-wide knockout (KO) screening to identify host factors required for STx-induced cell death [24,25,26]. In these screens, many membrane trafficking genes were enriched including COG complex subunits and GARP complex subunits involved in endosomes and the TGN/Golgi apparatus, which are likely involved in both GSL glycosylation and STx retrograde transport [24,27]. These screens and another screen related to EHEC infection also demonstrated that LAPTM4A and TM9SF2 are critical factors for the biosynthesis of Gb3 [24,25,27]. Loss of *LAPTM4A* genes decreases endogenous Gb3 synthase activity by a post-transcriptional mechanism, whereas loss of *TM9SF2* genes disrupts localization of Gb3 synthase at the TGN. Thus, identification of new glycan regulators will lead to a better understanding of the harmonized glycosylation mechanisms.

Permanent cell lines derived from non-human primates have been invaluable tools especially in medical studies. For example, Vero cells, which were isolated from the kidney of the African green monkey (AGM) *Chlorocebus sabaeus* [28,29], are susceptible to various viruses and bacterial toxins and, therefore, have been widely used as a tool for microbiological studies and as a cell substrate for human viral vaccines. STx is also known as Vero toxin, which was named after its lethal cytotoxicity to Vero cells [30].

Whole-genome sequencing of several Vero cell sublines indicated that their nucleotide sequences are ~95% identical to that of the human genome [29,31], invoking that single-guide RNA (sgRNA) libraries targeting the human genome are applicable to genome-wide KO screening for Vero cells. In addition, because experiments of genome-wide screening with the same sgRNA library but with different cell lines often produce different candidate genes, human sgRNA library-based STx-resistance screening experiments for Vero cells may allow us to identify genes that were not recognized in previous experiments for human cultured cells. In the present study, we performed STx-resistance screening in Vero C1008 cells using the GeCKO v2 sgRNA library [32] and showed that SYS1, a Golgi trafficking protein, is crucial for the production of the STx receptor Gb3.

## 2. Results

### 2.1. Human CRISPR KO Library Is Applicable to Vero Cell Lines

**Figure 1:**
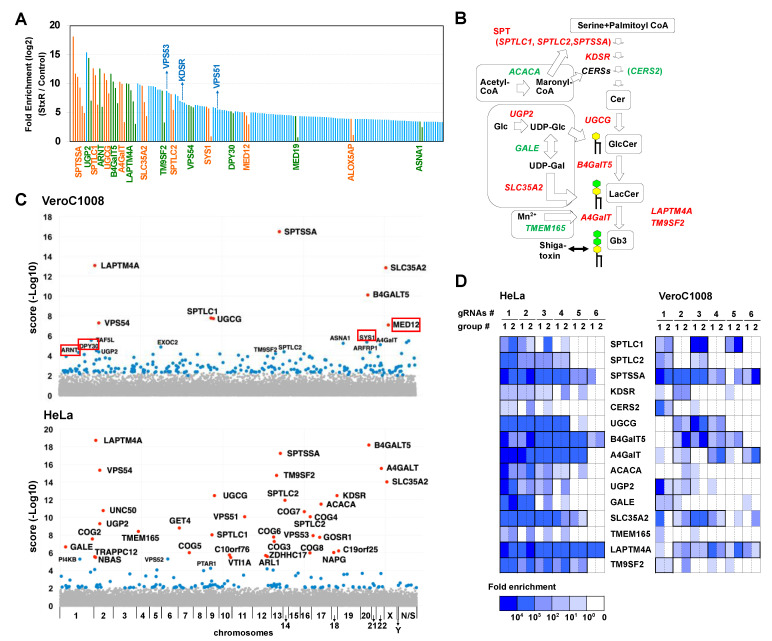
Identification of STx resistance genes in the genome-wide CRISPR screen. (**A**) Identification of sgRNAs enriched in the screen using Vero C1008 cells. Fold enrichment represents the mean of two independent experiments. Orange and green bars indicate that multiple sgRNAs were enriched in a gene, while blue bars indicate that a single sgRNA was enriched in a gene. The full raw data set is shown in Data S1A–C. (**B**) Gb3 biosynthetic pathway. Genes enriched in the Vero screen as well as the previous HeLa screen [24] are shown in red. Genes enriched only in the HeLa screen are shown in green. (**C**) Manhattan plots of the Vero screen and the HeLa screen. Scores were derived from the MAGeCK analysis shown in Data S1D. Red dots indicate FDR < 0.01. Blue dots indicate *p* < 0.01 but FDR > 0.01. (**D**) Fold enrichment of six sgRNAs in GSL-related genes enriched in the Vero screen compared with that of the HeLa screen. The heat map is representative of individual sgRNA enrichment (sg1–6) in two independent experiments (groups #1 and #2). See also Supplementary Materials.

We previously performed a genome-wide CRISPR KO screen in HeLa cells to identify host factors required for STx cytotoxicity [24]. In this screen, we used a subline of Vero cells, Vero C1008 (also called Vero E6), as the parent cells to determine whether the human lentivirus-based GeCKO v2 pooled library [32] could also be applied to monkey-derived cells. Two independent pools of sgRNA-expressed Vero C1008 cells were prepared by transducing with lentiviral libraries targeting 19,050 genes, and the cells were treated with STx1. The sgRNAs that integrated into the cellular genomes of surviving cells were analyzed with high-throughput sequencing, and the enrichment fold of each sgRNA after intoxication was calculated relative to untreated cells. We selected sgRNAs that were enriched in two separate screenings and also by more than 10-fold on average as STx-resistance sgRNA candidates (Figure 1A, the full raw data set is shown in Data S1–S3). Eighteen genes contained multiple enriched sgRNAs, which included sphingolipid-related genes such as *A4GalT* (Gb3 synthase), *B4GalT5* (LacCer synthase), *UGCG* (GlcCer synthase), *SPTLC1*, *SPTLC2*, *SPTSSA* (serine palmitoyltransferase subunits), *UGP2* (UDP-glucose synthase), and *SLC35A2* (UDP-galactose transporter) (Figure 1B). Previous screens identified *LAPTM4A* and *TM9SF2* as critical factors for the biosynthesis of Gb3 [24], and these genes were also enriched in this screen.

The degree of gene enrichment in the Vero screen was then compared with that in the previous HeLa screen using Model-based Analysis of Genome-wide CRISPR-Cas9 Knockout (MAGeCK) [33] (Figure 1C and Data S4). The number of genes enriched in the Vero screen was not as high as that in the HeLa screen. In particular, membrane trafficking genes (e.g., COG complex subunits) that had been moderately enriched in the HeLa screen were buried in the noise of irrelevant genes in this screen. The number of enriched sgRNAs of sphingolipid-related genes was also fewer in this screen (Figure 1D and Data S5, see in “Discussion” section). Nevertheless, the enrichment of most genes essential for receptor biosynthesis indicated that the Vero cell lines can be used for human KO library screening if high comprehensiveness is not desired.

### 2.2. Disruption of the SYS1 Gene Confers Resistance to STx-Induced Cell Death in Vero Cells

**Figure 2:**
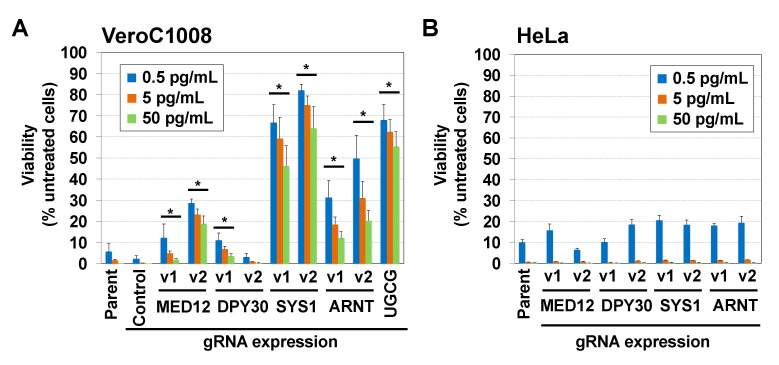
Unique genes enriched in the Vero screen. The reproducibility of STx resistance conferred by individual sgRNAs. Each sgRNA was lentivirally transduced into CAS9-expressed (**A**) Vero C1008 and (**B**) HeLa cells. The cells were treated with STx at the indicated concentrations (boxes) for 3 days. Viability was estimated using an MTT assay and is expressed as a percentage of the MTT value (OD_570_) in the absence of STx. “Parent” indicates non-transduced Vero C1008#5CAS9#6 cells and HeLa CAS9#W7 cells. “Cont” indicates irrelevant sgRNA (targeted to *CD11b*)-expressed cells. The percentages shown are the mean percentages ± SD obtained from three independent experiments. The Holm–Bonferroni corrected *t*-test was used for multiple comparisons. * Asterisks denote statistical significance.

A comparison of the two screens showed that several genes were enriched only in the Vero screen (Figure 1C). Among the genes, four remarkable genes, SYS1 Golgi trafficking protein (*SYS1*), mediator complex subunit 12 (*MED12*), Aryl hydrocarbon receptor nuclear translocator (*ARNT*), and Dpy-30 histone methyltransferase complex regulatory subunit (*DPY30*), were selected for validation of the results. Two sgRNAs for these genes were individually transduced into Vero C1008 cells and HeLa cells by lentiviruses to study their effect on STx-induced cytotoxicity. All but one sgRNA for *DPY30* conferred resistance to STx in Vero C1008 cells to a greater or lesser extent but not in HeLa cells (Figure 2A,B), indicating the reproducibility of both screens. We focused on SYS1 because both sgRNAs for SYS1 conferred the greatest resistance to STx among the four genes, and its role in STx-induced cell death is as yet unknown.

**Figure 3:**
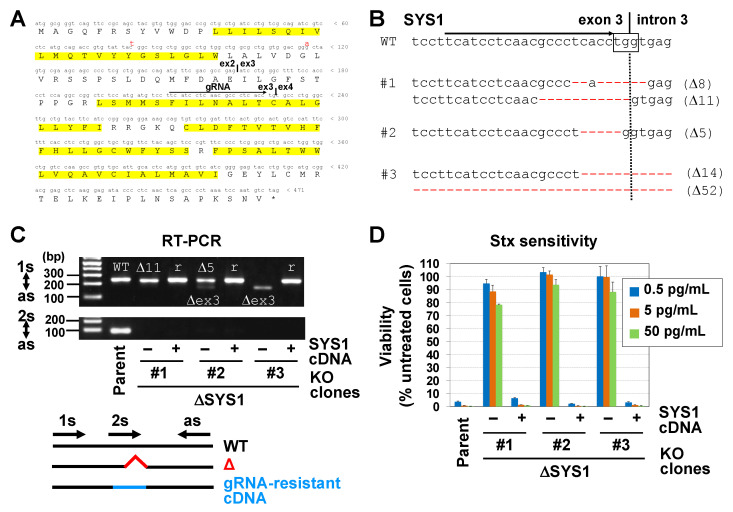
Resistance to STx in Vero C1008 *SYS1*-KO cells. (**A**) The open reading frame of human *SYS1* cDNA. Red letters indicate AGM *SYS1* cDNA sequences. Yellow shadows indicate predicted transmembrane domains. (**B**) Construction of Vero C1008 *SYS1*-KO cell clones (ΔSYS1#1–3). An arrow indicates the sgRNA-targeted sequence. Red letters in sequences are indicative of deletion mutations, which caused frameshifts shown at the right side of the sequences. Boxes indicate protospacer adjacent motif (PAM) sequences. Further information about the deletion sequences in clone 3 is shown in Figure S1A. (**C**) *SYS1* transcript analyses in ΔSYS1 cells and *SYS1*-rescued cells. RT-PCR analyses of *SYS1* mRNA in parent cells, ΔSYS1 cells (“−”: cDNA non-introduction), and *SYS1*-rescued cells (ΔSYS1/SYS1) (“+”: gRNA-resistant *SYS1* cDNA introduction) were performed using two pairs of primers. A pair of primers (s1 and as) amplified all *SYS1* transcripts, whereas another pair of primers (s2 and as) amplified only non-mutated transcripts but not mutated transcripts and gRNA-resistant cDNA. Sequence results of the PCR fragments that were amplified using the primers “s1” and “as” are shown in Figure S1. (**D**) STx sensitivity in ΔSYS1 cells and ΔSYS1/SYS1 cells. Cells were treated with STx1 at the indicated concentrations. Viability was estimated as described in Figure 1B and is expressed as the mean percentage ± SD obtained from three independent experiments.

SYS1 is a multi-spanning membrane protein mainly localized in the Golgi apparatus [34] (Figure 3A). To confirm resistance to STx caused by loss of SYS1, *SYS1* KO cell clones were generated using the CRISPR/CAS9 system. One of the sgRNAs contained in the library was selected and transduced into Vero C1008 cells by lentiviruses, and three clones were isolated. Genome sequence analysis demonstrated that the coding regions within exon 3 of *SYS1* had been deleted to cause frameshifts, or the exon 3–intron 3 junctions had been disrupted to cause a splicing error in all alleles of the three KO cell clones (Δ*SYS1*#1–3) (Figure 3B and Figure S1A). RT-PCR and sequence analysis confirmed that *SYS1* transcripts had been frameshifted in all clones (#1: Δ11 and Δex3 (Δ68), #2: Δ5 and Δex3, #3: Δex3) (Figure 3C and Figure S1B). All KO cell clones were highly resistant to STx-induced cell death (Figure 3D). To eliminate the possibility of off-target effects, sgRNA-resistant *SYS1* cDNA was introduced into the KO cells to prepare cDNA-rescued cells (Figure 3C and Figure S1). The cDNA-rescued cells fully restored STx sensitivity, confirming that the phenotype was due to the disruption of *SYS1* (Figure 3D). It should be noted that all KO cell clones changed their morphology from epithelial-like to fibroblast-like, and the morphology was also restored by *SYS1* cDNA introduction (Figure S2).

### 2.3. Loss of SYS1 Causes Defects in Glycan Biosynthesis

**Figure 4:**
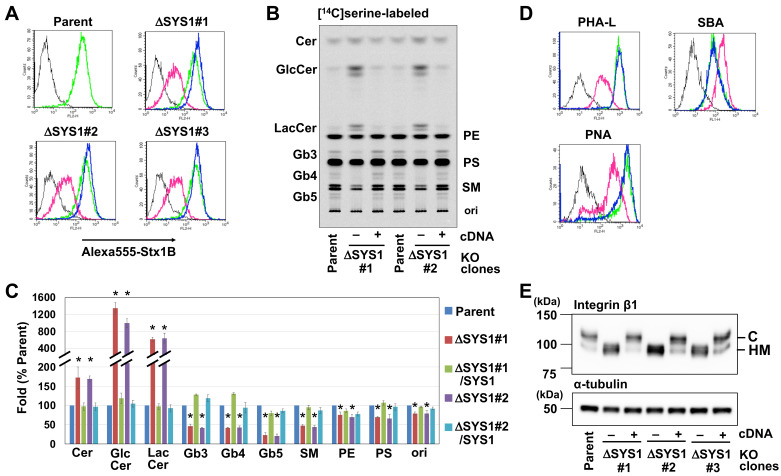
Glycan defects in ΔSYS1 cells. (**A**) Surface binding of STx on ΔSYS1 cells and ΔSYS1/SYS1 cells. Cells were stained with (green, magenta, and blue lines) or without (black line) Alexa555-labeled STx1 B subunit (Alexa555-STx1 B) and analyzed using FACS. Green lines indicate staining in parent cells. Black and magenta lines indicate staining in ΔSYS1 cells. Blue lines indicate staining in ΔSYS1/SYS1 cells. (**B**) GSL metabolic analysis of ΔSYS1 cells and ΔSYS1/SYS1 cells. Cells were labeled with [^14^C]serine, and labeled lipids were separated on a TLC plate. (**C**) Quantification of labeling experiments shown in B. The relative amount of each [^14^C]serine-labeled lipid is expressed as a percentage of band intensity in parent cells and is representative of the mean percentage ± SD obtained from three independent experiments. The Bonferroni corrected *t*-test was used for multiple comparisons. * *p* < 0.0125. (**D**) Surface binding of lectins on ΔSYS1 cells and ΔSYS1/SYS1 cells. Cells were stained with (green, magenta, and blue lines) or without (black line) FITC-conjugated lectins and analyzed using FACS. Green lines indicate staining in parent cells. Black and magenta lines indicate staining in *SYS1*-KO (ΔSYS) cells. Blue lines indicate staining in *SYS1*-rescued cells. (**E**) Western blot analysis of Integrin β1 in ΔSYS1 cells and ΔSYS1/SYS1 cells. Cell lysates were immunoblotted with anti-integrin β1 antibody (top) and anti-α-tubulin (bottom). C indicates complex-typed glycoproteins, and HM indicates high mannose-typed glycoproteins.

Flow cytometry demonstrated that cell surface binding of STx B-subunit was moderately reduced in all *SYS1*-KO cell clones, suggesting that the receptor Gb3 was reduced by the disruption of *SYS1* (Figure 4A). To determine whether Gb3 biosynthesis was affected, we metabolically labeled lipids with [^14^C] serine and [^14^C] galactose and analyzed the labeled lipids using TLC and radioactive imaging (Figure 4B and Figure S3A). [^14^C] serine-labeled lipids were quantified and graphed as a percentage of respective sphingolipid species in the parent cells (Figure 4C) as well as a percentage of total labeled sphingolipids in the parent cells (Figure S3B). Relative to parent cells, *SYS1*-KO cells had reduced levels of Gb3 (46.6 ± 4.5% in clone 1 and 41.2 ± 1.7% in clone 2) and subsequently synthesized globo-series GSLs (Gb4: 41.9 ± 1.5% in clone 1 and 42.3% ± 4.7% in clone 2; Gb5: 22.7% ± 6.4% in clone 1 and 20.8% ± f4.8% in clone 2). Instead, the KO cells had much higher levels of LacCer, the direct precursor of Gb3 (614.4 ± 42.9% in clone 1 and 638.2 ± 116.7% in clone 2) and GlcCer (1339.0% ± 133.4% in clone 1 and 993.2% ± 105.8%). The GSL alteration was also confirmed by a pulse-labelling experiment with [^14^C] galactose (Figure S3A). Introduction of SYS1 cDNA into *SYS1*-KO cells restored the GSL pattern including the amount of Gb3. These results indicate that SYS1 is required for proper biosynthesis of GSLs. Sphingomyelin (SM) and other phospholipids were also reduced in *SYS1*-KO cells; therefore, other lipid biosynthesis may be affected (see Discussion).

Next, to determine whether SYS1 is involved only in GSL biosynthesis or in other glycosylations, the effect of SYS1 on general glycosylation was analyzed by staining with three lectins: phytohemagglutinin-L (PHA-L), peanut agglutinin (PNA), and soybean agglutinin (SBA). PHA-l binds β1,6-*N*-acetylglucosamine (GlcNAc) branched complex-type N-glycan, whereas PNA binds galactose β1,3 *N*-acetylgalactosamine (Galβ1, 3GalNAc), which is often observed in core 1 *O*-glycans. Soybean agglutinin (SBA) binds terminal GalNAc including Tn antigen (GalNAc-serine/threonine), an immature *O*-glycan structure. Knockout of *SYS1* reduced the binding of PHA-L and PNA and increased the binding of SBA (Figure 4D), suggesting that SYS1 is required for maturation of both N- and O-glycan. Integrin β1 is known to have a lot of *N*-glycans [35], and Western blot analysis revealed that knockout of *SYS1* decreased the levels of the upper band, presumably containing mature complex-type glycans, and instead increased the levels of the lower band, presumably containing immature high mannose-type glycans. This result also supports the requirement of SYS1 for glycan maturation.

### 2.4. Loss of SYS1 Perturbs Golgi Morphology and TGN Distribution

**Figure 5:**
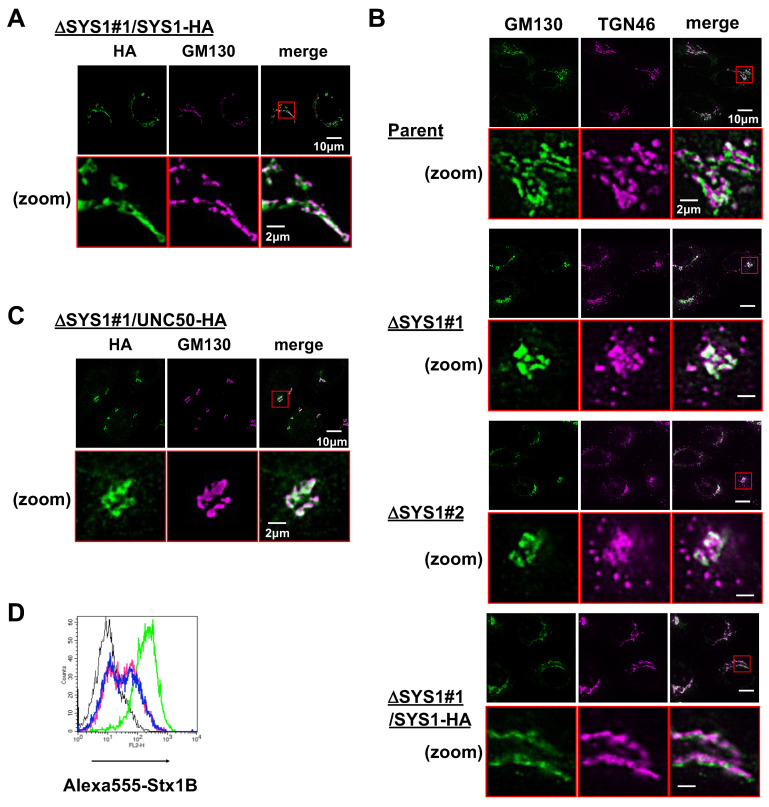
Perturbation of TGN46 distribution and Golgi morphological changes in ΔSYS1 cells. (**A**) Intracellular localization of SYS1. ΔSYS1/SYS-1-HA cells were stained with rat anti-HA antibodies (Abs) and mouse anti-GM130 (*cis* Golgi marker) Abs, followed by Alexa Fluor 488-conjugated anti-rat Abs (green) and Alexa Fluor 594-conjugated anti-mouse Abs (magenta). Scale bars, 10 µm and 2 µm (zoom). (**B**) Effect of *SYS1* KO on localization of TGN46. Parent cells, ΔSYS1 cells, and ΔSYS1/SYS-1-HA cells were stained with mouse anti-GM130 Abs and sheep anti-TGN46 (TGN marker) Abs, followed by Alexa Fluor 488-conjugated anti-mouse Abs (green) and Alexa Fluor 594-conjugated anti-sheep Abs (magenta). Scale bars, 10 µm and 2 µm (zoom). (**C**) Effect of *UNC50* cDNA on alteration of Golgi morphology by *SYS1* KO. ΔSYS1/UNC50 cells were stained with rat anti-HA antibodies (Abs) and mouse anti-GM130 (*cis* Golgi marker) Abs, followed by Alexa Fluor 488-conjugated anti-rat Abs (green) and Alexa Fluor 594-conjugated anti-mouse Abs (magenta). Scale bars, 10 µm and 2 µm (zoom). (**D**) Surface binding of STx on ΔSYS1/UNC50 cells. Cells were stained with (green, magenta, and blue lines) or without (black line) Alexa555-labeled STx1 B subunit (Alexa555-STx1 B) and analyzed using FACS. Green lines indicate staining in parent cells. Black and magenta lines indicate staining in ΔSYS1 cells. Blue lines indicate staining in ΔSYS1/UNC50 cells.

HA-tagging at the C-terminus of SYS1 (SYS1-HA) did not affect its function, and expression of SYS1-HA also restored STx binding in *SYS1*-KO cells (Figure S3C). SYS1-HA was localized to the Golgi apparatus as reported previously (Figure 5A) [36]. General glycosylation defects in *SYS1*-KO cells, which are shown in Figure 4, suggest that SYS1 affects the Golgi function. Fluorescent microscope analysis demonstrated that two Golgi-related phenotypes were observed in both *SYS1* KO clones. First, knockout of *SYS1* changed the staining pattern of GM130, a *cis* Golgi marker, from elongated layered structures to aggregated structures (Figure 5B), indicating that SYS1 affects Golgi morphology. Second, TGN46, a *trans*-Golgi network marker, was aligned with GM130 in parent cells, whereas KO cells had punctate staining of TGN46, which was not merged with GM130. Together with the previous reports in regards to retrograde trafficking of SYS1 [34,36,37], this result indicates that SYS1 is involved in the delivery of retrograde cargos to TGN and that knockout of *SYS1* fails to recruit some TGN46-containing vesicles.

### 2.5. SYS1 and UNC50 Are Functionally Different

UNC50 is a multi-pass membrane protein involved in retrograde transport from endosomes to TGN [38]. In contrast to *SYS1*, *UNC50* gRNAs were enriched in the HeLa screen [24] but not in the Vero screen. One possible reason why SYS1 and UNC50 were enriched only in one of the screens, respectively, is that SYS1 and UNC50 may be functionally redundant. Next, C-terminal tagged UNC50 was introduced into the *SYS1* KO cells to see whether UNC50 complements the dysfunction by knockout of SYS1. UNC50 was localized to the Golgi/TGN and aligned with GM130 as SYS1 (Figure 5C). However, the introduction of UNC50 did not recover the reduced STx-binding and the Golgi morphological defect in the *SYS1* KO cells, indicating that UNC50 is functionally distinct from SYS1 (Figure 5C,D).

## 3. Discussion

In this study, we screened the genes required for STx-induced cell death in Vero C1008 cells. The enriched genes in this screen included enzyme genes essential for Gb3 biosynthesis and Gb3-regulation including *LAPTM4A* and *TM9SF2*, which had been isolated in the previous screens [24,25,26,27]. This result indicates that the Vero cell line can be used for screening using a human CRISPR KO library. However, there was some dissatisfaction in terms of comprehensiveness because the number of enriched genes in the Vero screen was less than that in the HeLa screen. Moderately enriched genes in the HeLa screen, which were mainly intracellular transport genes, could not be enriched in the Vero screen except for Vps54, a GARP complex subunit (Figure 1C). A comparison of the gRNA target sequences for the GSL enzyme genes in the human KO library with the corresponding AGM DNA sequences showed that some sgRNAs had a different base than the AGM sequences (19 sgRNAs in 72 sgRNAs) (Data S5). Some of these different sgRNAs were not enriched in the Vero screen unlike the HeLa screen. However, some other sgRNAs were not enriched even though their bases were completely matched to the Vero genomic sequences. Therefore, differences in human and monkey sequences may not be the only reason for low enrichment, and differences in cell line characteristics such as genome editing efficiency and cell factor dependence may also reduce the comprehensiveness of screens. Vero cells are still the cell model of first choice for a variety of viral infections [39,40]. Recently, an African green monkey specific sgRNA library has been developed and applied to Vero cells to search for host factors required for infection of severe acute respiratory syndrome-related coronavirus 2 (SARS-CoV-2) [41]. However, human-specific sgRNA libraries are already in wide use around the world; therefore, the combination of Vero cells and a human CRISPR KO library would also contribute to the search for host factors in viral infections together with the monkey specific library.

Several genes were uniquely enriched in the Vero screen, and we focused on *SYS1* because lentiviral *SYS1* sgRNAs conferred a stronger resistance to STx. The isolated *SYS1* KO cell clones showed strong resistance to the toxin, and cell surface binding of STx was reduced in these cells; therefore, one of the causes of STx-resistance in KO cells is supposed to be the reduction of the receptor Gb3. SYS1 is a Golgi trafficking protein shared from yeast to vertebrates [34,36] and is often used as a *trans*-Golgi apparatus marker in yeast [42]. SYS1 is known to be involved in membrane trafficking around the Golgi apparatus. However, it is less reported how the loss of SYS1 affects Golgi functions including glycan biosynthesis, although SYS1 is reported to regulate the cell surface presentation of ADAM10, the receptor of α-hemolysin produced by *Staphylococcus aureus* [43]. SYS1 targets ADP-ribosylation factor-related protein ARFRP1 (also known as ARL3) to the Golgi apparatus [36,44]. ARFRP1 promotes the recruitment of GARP complex as well as some golgins to the TGN, indicating that the SYS1-ARFRP1 complex regulates endosome-derived carrier tethering to the TGN [37]. In this screen, not only SYS1 but also ARFRP1 was enriched (Figure 1C), suggesting that SYS1/ARFRP1-mediated vesicle transport is involved in GSL biosynthesis.

GSL analysis of the KO cells demonstrated two phenotypes: a decrease in Gb3 along with the subsequently synthesized globo-series GSLs and a significant increase in GlcCer and LacCer. In HeLa cells, knockout of *A4GalT* (Gb3 synthase) simply increases the precursor LacCer but not GlcCer [24]. Therefore, knockout of *SYS1* is thought to affect not only the Gb3 synthesis step in the TGN but also the GlcCer synthesis step in the Golgi apparatus. A4GalT is predominantly localized to the TGN [16,17,18]. This study demonstrated that knockout of SYS1 perturbed the distribution of TGN46 and showed punctate staining of TGN46 out of alignment with the Golgi apparatus (Figure 5B). In our previous studies, knockout of *TM9SF2* or overexpression of TMBIM family proteins also reduced Gb3 along with punctate structures of TGN46 in HeLa cells [17,24]. Therefore, SYS1 is thought to contribute to Gb3 biosynthesis by proper membrane transport to the TGN. The effect of SYS1 on GSL biosynthesis does not seem to be limited to A4GalT alone. Knockout of SYS1 increased not only LacCer but also GlcCer, which was 10 times more abundant than in the parent cells. GlcCer accumulation is also observed following Golgi transport perturbation in conserved oligomeric Golgi (COG) complex-defective cells [45] and Brefeldin A (BFA)-treated cells [46,47,48]. COG complex is involved in intra-Golgi retrograde trafficking, which is responsible for the localization of Golgi glycosyltransferases and affects glycosylation of both glycoproteins and GSLs [21,22,23]. BFA inhibits COP I vesicle transport and causes glycosylation defects by fusion of the Golgi membrane to the ER [49,50,51]. The GlcCer synthase UGCG is thought to be localized to the *cis*-Golgi apparatus, although localization of endogenous UGCG has not been clarified yet. Perturbation of the intra-Golgi transport or the ER-Golgi transport may change the localization of UGCG, resulting in easier access to ceramides, which are mainly synthesized at the ER. Knockout of *SYS1* also reduced SM, possibly because the preferential utilization of ceramides by UGCG competes with SM synthesis, resulting in the reduction of SM. Together with the result that knockout of *SYS1* also perturbed glycosylation on glycoproteins including integrin β1 as well as Golgi morphology (Figure 4D,E and Figure 5E), SYS1 must affect general glycan biosynthesis by regulation of intra-Golgi membrane transport in addition of endosome–TGN retrograde transport. It is unclear why the synthesis of phospholipids including phosphatidylserine (PS) and phosphatidylethanolamine (PE) was reduced in *SYS1* KO cells. Previously, BFA treatment reduced the synthesis of these phospholipids in HeLa cells [48]. Therefore, Golgi trafficking defects may inhibit the synthesis of PS and PE.

*SYS1* was enriched only in the Vero screen, and lentiviral introduction of *SYS1*-sgRNA did not confer resistance to STx in HeLa cells. This was consistent with the previous result that *SYS1* was not enriched in the HeLa screen. We failed to isolate *SYS1* KO cell clones in HeLa cells although we confirmed that the SYS1 sgRNA used (v2 shown in Figure 2) was able to cause mutations. Previously, SYS1 was isolated as a cell cycle regulator by genome-wide screening using an RNA interference (RNAi) library, and it was shown that depletion of SYS1 by RNAi in HeLa cells delayed mitotic progression [52]. Therefore, one of the reasons why *SYS1* was not enriched in the HeLa cells could be that *SYS1* affects cell proliferation in HeLa cells. Another reason could be that genes functionally redundant to *SYS1* are expressed in HeLa cells. UNC50 is a multi-spanning membrane protein and is known to be involved in retrograde trafficking from endosomes to TGN [38]. The *UNC50* gene was enriched in the HeLa screen but not in the Vero screen. Furthermore, *UNC50* is enriched in not only GSL-related screens but also glycoprotein- and proteoglycan-related screens [53,54,55]. These features of UNC50 are similar to SYS1. However, *UNC50* did not compensate for the defect of *SYS1* including the Gb3 reduction, the Golgi shape, and cell shape (Figure 5B,C). Therefore, both proteins have different functions in retrograde trafficking. However, we cannot rule out the possibility that SYS1- and UNC50-dependent trafficking pathways independently contribute to the transport of common cargo molecules. Differences in the dependence of the SYS1-dependent pathway and other similar pathways among cell lines need to be clarified in the future.

In summary, we performed a genome-wide loss-of function screen to identify genes that conferred resistance to STx in Vero cells. The results of this screen indicated that a human CRISPR library is applicable for Vero cells, despite the differences in species. Among the isolated factors, SYS1 played an important role in the regulation of GSL biosynthesis, and the knockout of *SYS1* resulted in reduced Gb3 and accumulated GlcCer as well as defective glycosylation of membrane proteins. Together with the result of disrupted localization of TGN46 in the KO cells, SYS1 was required for proper glycosylation.

## 4. Materials and Methods

### 4.1. Cell Culture, Antibodies, and Reagents

Vero C1008 cells (CRL-1586) were obtained from the American Type Culture Collection (ATCC). The cells were serially diluted to isolate a clone (Vero C1008 #6) highly sensitive to STx, which was used as a parent cell in this study. Vero C1008 and subclones including its KO mutants and transfectants were maintained in Minimum Essential Medium (MEM) containing 10% heat-inactivated fetal bovine serum (FBS). The HeLa-mCAT#8 cells, which express mouse cationic amino acid transporter 1 (which serves as a mouse ecotropic retroviral receptor) [17], were maintained in Dulbecco’s modified Eagle’s medium (DMEM) containing 10% FBS. 293FT cells (Thermo Fisher, Rockford, IL, USA) for lentivirus production were maintained in DMEM containing 10% FBS with non-essential amino acids and sodium pyruvate. Plat-E cells [56] for retrovirus production were maintained in DMEM containing 10% FBS.

Purchased antibodies (Abs) were as follows: mouse anti-GM130 IgG (BD Transduction Laboratories, San Diego, CA, USA), sheep anti-TGN46 Abs (Serotec, Kidlington, UK), and rat anti-HA IgG (3F10, Roche Diagnostics, Mannheim, Germany). Alexa-conjugated secondary antibodies were purchased from Thermo Fisher, except for the Alexa-594 donkey anti-sheep F(ab’)_2_ fragment, which was purchased from Jackson ImmunoResearch (West Grove, PA, USA). FITC-conjugated PHA-L and SBA were purchased from Vector Laboratories (Burlingame, CA, USA). FITC-conjugated PNA was purchased from J-Oil Mills (Tokyo, Japan).

3-(4,5-Dimethylthiazoyl-2-yl)-2,5-diphenyltetrazolium bromide (MTT) and puromycin were purchased from Sigma-Aldrich. Thin-layer chromatography (TLC) and high-performance thin-layer chromatography (HPTLC) plates (Silica Gel 60) were purchased from Merck (Darmstadt, Germany). l-[U-^14^C]Serine (174 mCi/mmol) was purchased from Moravek (Brea, CA, USA). d-[1-^14^C]Galactose (56 mCi/mmol) was purchased from GE Healthcare (Buckinghamshire, UK). Geneticin was purchased from Nacalai (Kyoto, Japan). Polyethylenimine Max (PEI-Max) was purchased from Polysciences Inc (Warrington, PA, USA). Shiga toxin 1 (STx1) derived from *E. coli* O157:H7 was a kind gift from Dr. Kiyotaka Nishikawa (Doshisya University, Kyoto, Japan) [57]. Preparation of fluorescent STx1 B subunit (Alexa555-STx1 B) was conducted as described previously [17]. The human Genome-scale CRISPR Knock-Out (GeCKO) v2.0 library in the lentiGuide-Puro plasmid (65386 single-guide RNAs (sgRNAs) in library A and 58031 sgRNAs in library B) and the lentiCAS9-Blast plasmid (two vector lentiviral GeCKO system) were obtained from Addgene [32]. Primers used in this study are described below.

### 4.2. Isolation of the Cas9-Expressing Vero C1008 Cell Clone for CRISPR Screen

293FT cells were transfected with a lentiCAS9-Blast plasmid and ViraPower packaging plasmids (Thermo Fisher) to produce lentivirus for CAS9 expression (CAS9-lentivirus). Subsequently, Vero C1008#6 cells were infected with the CAS9-lentivirus, and the CAS9-expresing cells were grown in the presence of 15 µg/mL blasticidin. The clone with the highest genome-editing efficiency was selected as the parent cell clone (Vero C1008#6 CAS9#5) for the CRISPR screen.

### 4.3. Preparation of sgRNA-Expressing Cell Libraries

Four independent lentiviral pools (A-1, A-2, B-1, and B-2) of the GeCKO v2.0 library were prepared and stored as described previously [24]. Vero C1008#6 CAS9#5 cells (2.4 × 10^7^ cells) were infected with each lentivirus pool at a low MOI (about 0.2). Forty-eight hours after transduction, cells were selected with 7.5 µg/mL puromycin for 5 days to prepare sgRNA-expressing Vero C1008 cell libraries.

### 4.4. CRISPR Screen for STx1 Treatment

Approximately 2.4 × 10^7^ sgRNA-expressing cells from each cell library (A-1, A-2, B-1, and B-2) were plated at 24 h prior to treatment with 20 pg/mL STx1. Five days after the STx1 treatment, cells were cultured in the absence of STx1 for 3 days. Surviving cells were then re-plated and treated again with 50 pg/mL STx1. Eleven days after treatment, cells were trypsinized and frozen as cell pellets. For untreated controls, 1.2 × 10^7^ sgRNA-expressing cells in each cell library were cultured for the same period as STx1-treated cells with several passages, such that a minimum of 1.2 × 10^7^ cells was present in each passage.

### 4.5. Genomic DNA Sequencing

Analysis of genome-integrated sgRNAs was performed as follows [24]: genomic DNA from frozen cells was purified using the conventional phenol–chloroform method. Amplification of the genome-integrated sgRNA sequences by PCR was performed as follows, based on a previous method [58]. For the first PCR, 27 µg of genomic DNA from untreated cells or 13.5 µg of genomic DNA from STx-treated cells was used as a PCR template. For each sample (A-1, A-2, B-1, and B-2), eight separate 100 µL reactions were performed using PrimeStar GXL DNA polymerase (Takara, Otsu, Japan) and the following primers (nine forward primers and one reverse primer):

(Fw) 1stY1R1s0–8: CTACACGACGCTCTTCCGATCT (0–8 bp random sequence for increasing library complexity) TCTTGTGGAAAGGACGAAACACCG

(Rv) 1stY2as: GCCACTTTTTCAAGTTGATAACGGACTAG

Amplification was carried out over 25 cycles; 0.5 µL from each of the nine separate first PCR products was used as a template for the second round of PCR. For each sample, nine separate 20 µl PCR reactions were performed using the following primers:

(Fw) 2nd P5R1s: 

AATGATACGGCGACCACCGAGATCTACA**C** **TCTTTCCCTACACGACGCTCTTCCGATCT**

(Rv) 2nd P7Y2as: CAAGCAGAAGACGGCATACGAGAT (CC (A-1), or TT (A-2), or AA (B-1), or GG (B-2) as barcodes for multiplexing of different samples)

GCCACTTTTTCAAGTTGATAACGGACTAG

Illumina adaptor sequences (P5 and P7 respectively) are underlined, whereas the sequence primer site for MiSeq sequence analysis is indicated by bold font. Amplification was carried out over 15 cycles. The resulting nine amplicons in each sample were mixed, and the gel was extracted using SYBR Gold (Thermo Fisher). The extracted DNA was then quantified using a Quantus fluorometer (Promega, Madison, WI, USA), as well as running an agarose gel with a 100 bp quantifiable DNA Ladder (NEB, Ipswich, MA, USA), and then equal amounts of each sample (A-1, A-2, B-1, and B-2) were mixed. The DNA concentration of the mixture was adjusted for sequencing analysis. PhiX Control Kit v3 (Illumina, San Diego, CA, USA) was added to the sample at approximately 20% concentration. MiSeq Reagent Kit v3 (Illumina) was used for MiSeq sequencing (Illumina).

### 4.6. Data Processing and Analysis

Data processing and analysis were performed as follows [24]: To perform demultiplexing of fastq sequence data, total raw read sequences were divided into each sample with barcode sequences of “AA”, “CC”, “GG”, and “TT” using an in-house program. The adapter sequences were removed using a skewer program (version 0.1.126) [59] with the following parameters: minimum read length = 10 mer, maximum read length = 30 mer, and lowest mean quality value = 19 sanger quality score. To extract high-quality sgRNA sequences, sequences with a Phread quality score less than 20 were excluded using the “split_libraries_fastq.py” (version 1.9.1) function of the QIIME program [60]. The number of sgRNA sequences was calculated with “sort” and “uniq” of the unix command program, followed by normalization with the following formula: normalized reads per sgRNA = reads per sgRNA/total reads for all sgRNAs in sample × 10^7^ (Data S1 and S2). Fold enrichment was calculated using the following formula: Fold enrichment = normalized reads in STx-treated sample/normalized reads in untreated sample. When the normalized read in the untreated sample was 0, fold enrichment was calculated by setting 0 to 1. First, identification of essential genes that were closely related to STx1 interaction was performed using the MAGeCK program (version 0.5.7) [33] to analyze normalized sgRNA count data (Data S4). In this program, 640 genes contained at least one significantly different sgRNA. For stricter selection of hit sgRNAs, the sgRNAs representing more than 1-fold enrichment in both independent cell libraries (A-1 and A-2, or B-1 and B-2) were selected as STx resistance sgRNA candidates (Data S3), and the fold enrichment of these candidates is graphed in Figure 1A. Note that the selected sgRNAs were all statistically significantly enriched, which was demonstrated using the MAGeCK program (Data S4). The statistical data of enriched genes analyzed by MAGeCK was visualized as a Manhattan plot using Plotly JavaScript Open Source Graphing Library (plotly.js version 1.50.0).

### 4.7. Sequences of AGM Genome and Transcripts

AGM genome sequences were taken from RefSeq Genome Database of the NCBI. Vero transcript sequences were taken from the Sequence Read Archive of the NCBI in which our group had deposited the RNA-seq short reads previously (accession number: DRA002256) [31].

### 4.8. Preparation of Lentiviruses for gRNA Expression

LentiGuide-Puro (Addgene), a lentiviral plasmid for sgRNA expression, was cleaved with BsmBI, and a 20-mer guide sequence was ligated into the site. The sequence of the 20-mer guide sequence was confirmed using an ABI3100 sequencer. 293FT cells (1 × 10^6^ in 6-well plates) were transfected with 0.6 µg of the lentiviral plasmids and 2.4 µg of ViraPower packaging plasmids (Thermo Fisher) using PEI-Max. After 24 h, media was changed and the cells were cultured for an additional 24 h. Subsequently, the culture media containing secreted lentiviruses was filtered using a 0.45 µm bottle top filter and frozen as lentiviral pools. The sgRNA sequences used in this study are described below.

### 4.9. Construction of CRISPR KO Cell Lines

Vero C1008#6 CAS9#5 cells (1 × 10^5^ cells/well in 6-well plates) and HeLa CAS9#W7 cells (5 × 10^4^ cells/well in 6-well plates) [24] were infected with lentiviruses for sgRNA expression. Forty-eight hours after infection, cells were selected with 7.5 µg/mL puromycin. The selected cell pools are shown in Figure 2. For the preparation of *SYS1* KO cells, the sgRNA-expressing cells were cloned by serial dilution. Indel analysis for KO confirmation was performed as previously described [61]. Briefly, trypsinized cells were heated in TE buffer followed by vortexing to use as a template of genomic PCR. PCR was performed with PrimeSTAR GXL, and the PCR products were sequenced using an ABI3130 Genetic Analyzer (Thermo Fisher).

### 4.10. RNA Isolation, Reverse Transcription (RT)-PCR, and Sequencing

Total RNA was isolated using TRIzol Reagent as per the manufacturer’s instructions (Thermo Fisher). RT was performed using the ReverTra Ace qPCR RT Master Mix (ReverTra Ace, Toyobo) as per the manufacturer’s instructions, including a DNase I treatment step. The RT products were used as PCR templates (primer pair #1: 1s and as, primer pair #2: 2s and as), and the amplified PCR products were run on a gel and sequenced.

### 4.11. Preparation of Retroviruses for cDNA Expression and Stable Transfectants

Human sgRNA-resistant *SYS1* cDNA was purchased from Eurofins Genomics (Tokyo, Japan). The sgRNA-resistant *SYS1* cDNA in the pEX-A2J2 plasmid was cleaved with restriction enzymes and inserted into pMXs-IN, a retroviral vector plasmid (pMXs-IN–SYS1). For HA-tagging at the C-terminus, the sgRNA-resistant *SYS1* cDNA in the absence of stop codon was amplified by PCR using pMXs-IN–SYS1 as a template and a set of primers (pMX-Fw2 and SYS1 Xho-END as). The PCR fragment was digested with restriction enzymes and inserted into pMXs-IN along with a HA-tag sequence (pMXs-IN–SYS1-HA). Human *UNC50* cDNA in the absence of stop codon was prepared by PCR using HeLa cDNA as a template and a set of primers (UNC50 Bam-ATG s and UNC50 Xho-END as) and inserted into pMXs-IN with a HA-tag sequence (pMXs-IN–UNC50-HA). Preparation of retroviruses and infection of Vero C1008 *SYS1* KO cells were performed using the Plat-E system [56] with some modifications. Plat-E cells (8 × 10^5^ in 6-well plates) were transfected with 0.5 µg of pMXs-IN-based plasmids and 0.5 µg of pLP/VSV-G plasmid (Thermo Fisher) to produce amphotropic retroviruses. Vero C1008 *SYS1* KO cells (1 × 10^5^ cells/well in 6-well plates) were infected with the prepared retroviruses. Forty-eight hours after infection, cells were selected with 400 µg/mL geneticin. The selected cell pools were used.

### 4.12. Immunofluorescence Microscopy

Immunostaining was performed as described previously [53]: Cells were grown on a glass coverslip in a 6-well plate for 72 h. The cells were fixed with Mildform 10N (Wako, Osaka, Japan) for 20 min at room temperature. After washing twice with PBS, the cells were sequentially incubated at room temperature with 0.1 M NH_4_Cl in PBS for 20 min and with 0.1% Triton X-100 for 15 min. After washing twice with PBS, the cells were incubated with 3% bovine serum albumin (BSA) in PBS for 30 min. The cells were then incubated with rat anti-HA IgG, mouse anti-GM130 IgG, and sheep anti-TGN46 for 1 h. After washing three times with PBS, the cells were incubated with Alexa488- and Alexa594-conjugated secondary antibodies for 1 h. After washing three times with PBS, the coverslips were mounted on a Fluoromount (Diagnostic Biosystems, Pleasanton, CA, USA). The specimens were visualized using a wide-field fluorescence microscope, BZ-X700 (Keyence, Osaka, Japan), equipped with a Plan Apo VC 60x1.20 WI (water immersion) objective. A haze reduction function (condition 2), which applies a no-neighbor deconvolution algorithm to the captured image, was used to eliminate fluorescence blurring caused by scattered light and to capture clear images with high contrast.

### 4.13. Lysate Preparation and Western Blot Analysis

Cells were sonicated in sonication buffer (10 mM Hepes/NaOH (pH 7.4), 1 mM EDTA, 0.25 M sucrose, protease inhibitor cocktail) and subsequently mixed with Laemmli sodium dodecyl sulfate (SDS) sample buffer. Protein concentrations were determined using the Pierce BCA protein assay kit using BSA as a standard. Proteins were resolved by SDS-PAGE, transferred to PVDF membranes using the wet transfer method, and probed with specified antibodies. Antigen signals were detected using SuperSignal West Femto Maximum Sensitivity Substrate (Thermo Fisher) or Chemi-Lumi One L (Nacalai) and exposed to an X-ray film.

### 4.14. Metabolic Labeling of Glycolipids and TLC Analysis

Metabolic labeling experiments using l-[U-^14^C]serine and d-[1-^14^C]galactose including mild alkaline methanolysis were performed as described previously [48]. Cells (3 × 10^5^/well in a 6-well plate) were cultured overnight at 37 °C, and then cells were incubated with 22.2 kBq of l-[U-^14^C]serine or 7.4 kBq of d-[1-^14^C]galactose in Opti-MEM with 1% Nutridoma-SP (Roche) for 16 h. Cells were lysed with 0.1% sodium dodecyl sulfate (SDS), and lysates containing the same amount of protein were used for lipid extraction following the method of Bligh and Dyer [62]. For alkali-methanolysis to remove glycerolipids, dried lipids pulse-labeled with d-[1-^14^C]galactose were hydrolyzed with 0.1N KOH in methanol for 1 h at 40 °C. After neutralization with 0.1N HCl, the methanol layer was washed twice with *n*-hexane, and the lipids were extracted using the method of Bligh and Dyer. The lower fractions collected were dried under an N_2_ gas stream. Separation of lipids by TLC was performed using two methods as follows. Method 1: A TLC60 plate (20 cm × 20 cm), developing solvent: methyl acetate/n-propanol/chloroform/methanol/0.25% KCl = 50/50/50/20/18 [61] or Method 2: A HPTLC60 plate (10 cm × 20 cm), developing solvent: chloroform/methanol/0.25% CaCl_2_ = 65/35/8 [17]. Method 1 was used in Figure 4B, and Method 2 was used in Figure S3A. The radioactive lipids on TLC plates were visualized, and the intensity of each band was quantified using a Typhoon FLA 7000 (GE Healthcare, Buckinghamshire, UK). To compare the relative amounts of the lipids, the band intensity of each GSL in the parent cells was considered to be 100%.

### 4.15. FACS Analysis

Non-confluent cells were trypsinized and washed with culture medium and wash buffer (1% BSA) in PBS at 4 °C. Cells were incubated with 10 µg/mL Alexa-555 STx1 B subunits or FITC-lectins for 45 min on ice. After washing once with wash buffer, cells were analyzed using a FACSCalibur (BD Biosciences, Franklin Lakes, NJ, USA).

### 4.16. STx Treatment and cell Viability Assay

For treatment with STx1, cells (1–1.5 × 10^4^ cells/mL in 12-well or 24-well plates) were cultured overnight at 37 °C, and then treated with STx1 at the indicated concentrations for 3 days. An MTT assay was then performed as described previously to assess cell viability [17].

### 4.17. Statistical Analysis

In Figure 2A, the Holm–Bonferroni sequential correction [63] was used for 10 comparisons (10 gRNA-expressed cells to parent cells), with *p_i_* < 0.05/(10-i + 1), *p*_1_ (the smallest *p*) < 0.005 (0.05 divided by 10), and *p*_10_ (the largest *p*) < 0.05 (0.05 divided by 1). In Figure 4C, the Student’s *t*-test with Bonferroni correction was used, with *p* < 0.0125 (0.05 divided by 4) considered to be statistically significant in four comparisons (parent vs. other four samples).

### 4.18. Primers Used in This Study

Primers for indel analysis (*SYS1*)

SYS1 int2 s1: CTGCCGTACAGATGTAGGGAGGTATTATC

SYS1 int3 as1: GAAACAAGTGCCAGCCTGTCTCATG

Primers for constructing expression vectors (Underlines are indicative of restriction enzyme cutting sites)

pMX-Fw2: TAGACGGCATCGCAGCTTGG

SYS1 Xho-END as: ACCCTCGAGGACATTGGATTTAGGGGCTGAGTTG

UNC50 Bam-ATG s: ACCGGATCCAAGATGTTACCGAGTACTTCAGTG

UNC50 Xho-END as: ACCCTCGAGTTTCACTCTGTACTTATAGAAAGAAC

Primers for RT-PCR

hSYS1-RT 1s (Ex2): ATCGTCCTCATGCAGACCGTG

hSYS1-RT 2s (Ex3): ATCCTCAACGCCCTCACCTG

hSYS1-RT as (Ex4): GGACAGTGACAGTGAAATCCAGA

### 4.19. sgRNA Target Sequences

SYS1 v1: TGAGCTCCGTCCGCATGCAC

SYS1 v2: TCATCCTCAACGCCCTCACC

ARNT v1: GTGGAGGAGCCATTGTCCAG

ARNT v2: TGAATAGGCTGAGCTTTGTG

MED12 v1: CGTCAGCTTCAATCCTGCCA

MED12 v2: TAACTGCTCCCATAAGTACT

DPY30 v1: TTGCTGTGCTTGCAAAGGAA

DPY30 v2: TGTCCCTCCAGCATCTGCTC

UGCG (positive control): GGGCCGGGGGATGGCGCTGC

CD11b (negative control): AGGACTCTGAGAGCCATGGC

## Data Availability

The sgRNA data reported in this study have been deposited in the NCBI GEO and are available under accession number GSE169364.

## Supplementary Materials

The following are available online at https://www.mdpi.com/article/10.3390/ijms22094936/s1. Data S1. Read count, normalization and statistical analysis of sgRNA in GeCKO version 2 library kit Set A, related to Figure 1. Data S2. Read count, normalization and statistical analysis of sgRNA in GeCKO version 2 library kit Set B, related to Figure 1. Data S3. Summary of enriched genes in STx-resistant Vero cells, related to Figure 1. Data S4. MAGeCK analysis of STx-screens in Vero C1008 and HeLa cells, related to Figure 1. Data S5. Differences between gRNA and Vero sequences, related to Figure 1. Figure S1. SYS1 genome and transcript sequences in ΔSYS1 cell clones. Figure S2. Cellular morphological changes in ΔSYS1 cell clones. Figure S3. GSL defects in ΔSYS1 cells.
