# Supplementary material for: Identification of SYS1 as a Host Factor Required for Shiga Toxin-Mediated Cytotoxicity in Vero Cells
Source: Int J Mol Sci. 2021 May 6;22(9):4936. doi: 10.3390/ijms22094936 (PMC8124574; doi:10.3390/ijms22094936)
Supplement: Supplementary file 1 [file ijms-22-04936-s001.zip › Supplemantary FigureS1-S3 Sakuma et al.pdf]

A Genome

Parent

exon | intron

ctctccatgatgtccttcatcctcaacgccctcacctggtgagtatcaccagtttggcta (WT)

ΔSYS1#1

ctctccatgatgtccttcatcctcaacgccc--a------gagtatcaccagtttggcta (Δ8)

ctctccatgatgtccttcatcctcaac-----------gtgagtatcaccagtttggcta (Δ11)

ΔSYS1#2

ctctccatgatgtccttcatcctcaacgccct-----ggtgagtatcaccagtttggcta (Δ5)

ΔSYS1#3

ctctccatgatgtccttcatcctcaacgccct----t----------accagtttggcta (Δ14)

ctctcca--//---gcttttggg (Δ52)

B RNA

Parent

exon3 | exon4

ctctccatgatgtccttcatcctcaacgccctcacctgtgccctgggcttgctgtacttcatc (WT)

L S M M S F I L N A L T C A L G L L Y F I

ΔSYS1#1

ctctccatgatgtccttcatcctcaac-----------tgccctgggcttgctgtacttcatc (Δ11)

L S M M S F I L N C P G L A V L H

frameshift

75 irrelevant aa & STOP codon

ΔSYS1#2

ctctccatgatgtccttcatcctcaacgccct-----gtgccctgggcttgctgtacttcatc (Δ5)

L S M M S F I L N A L C P G L A V L H

frameshift

75 irrelevant aa & STOP codon

ΔSYS1#1, 2 & 3

exon2 | exon4

cgaagcagcccctcgctggaccagatgttcgacgccgagtgccctgggcttgctgtacttcatc (Δex3) (Δ68)

R S S P S L D Q M F D A E C P G L A V L H

frameshift

75 irrelevant aa & STOP codon

SYS1 gRNA-resistant cDNA

ctctccatgatgtcctttatttgaatgcgttgacgtgtgccctgggcttgctgtacttcatc (Silent mutaions)

L S M M S F I L N A L T C A L G L L Y F I

Figure S1. ***SYS1* genome and transcript sequences in ΔSYS1 cell clones**

(A) Genome sequences. An arrow indicates the sgRNA-targeted sequence. Red letters in sequences are indicative of deletion mutations, which caused frameshifts shown at the right side of the sequences. Boxes indicate protospacer adjacent motif (PAM) sequences.

(B) Transcript sequences. RT-PCR fragments amplified using a pair of primers (s1 and as), shown in Figure 3C, were sequenced. Red letters in RNA sequences are indicative of deletion mutations, which caused frameshifts shown at the right side of the sequences. Frameshifted amino acid sequences are shown in red letters. Related to Figure 3.

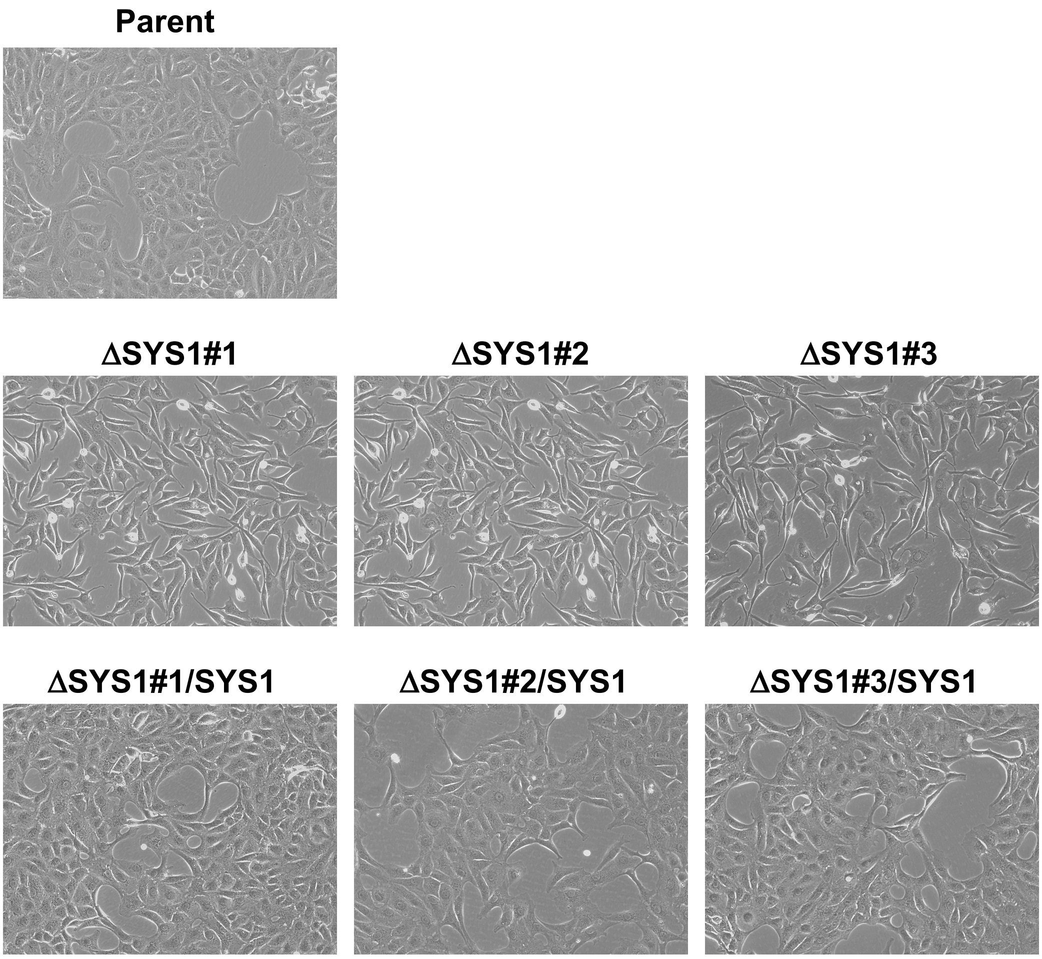

Figure S2. Cellular morphological changes in ΔSYS1 cell clones
Related to Figure 3.

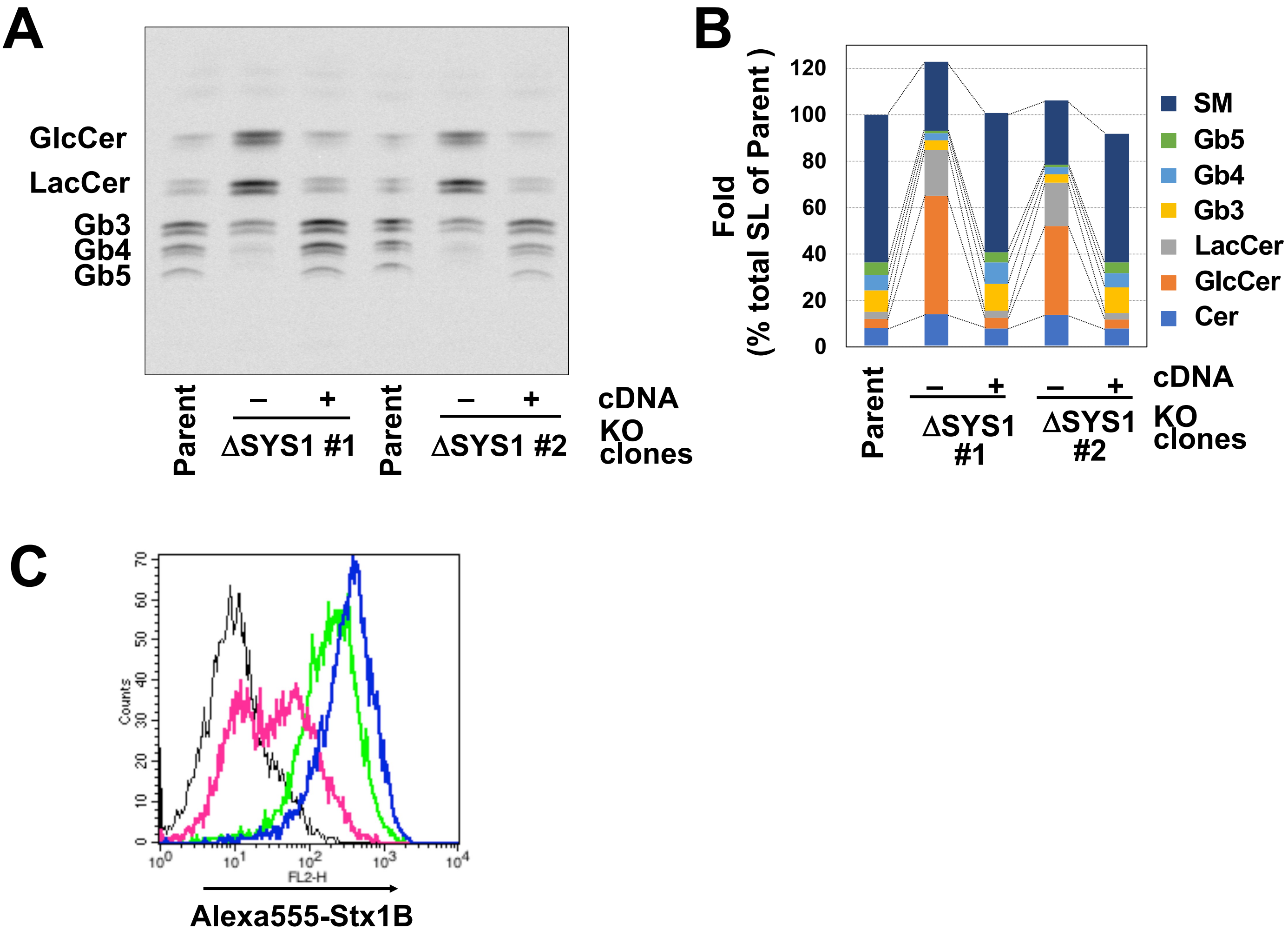

Figure S3. GSL defects in ΔSYS1 cells

(A) GSL metabolic analysis of ΔSYS1 cells and ΔSYS1/SYS1 cells. Cells were labeled with [^{14}C]galactose, and labeled lipids were separated on a TLC plate. Related to Figure 4.

(B) Quantification of labeling experiments shown in Figure 4B. The relative amount of [^{14}C]serine-labeled lipid is expressed as a percentage of band intensity derived from total sphingolipids in parent cells and is representative of the mean percentage from three independent experiments. Related to Figure 4.

(C) Surface binding of STx on ΔSYS1/SYS1-HA cells. Cells were stained with (green, magenta, and blue lines) or without (black line) Alexa555-labeled STx1 B subunit (Alexa555-STx1 B) and analyzed using FACS. Green lines indicate staining in parent cells. Black and magenta lines indicate staining in ΔSYS1 cells. Blue lines indicate staining in ΔSYS1/SYS1-HA cells. It should be noted that HA tagging at the C-terminus did not affect SYS1 functions of GSL regulation. Related to Figure 5.
